# Expression, purification, crystallization and X-ray data collection for RAS and its mutants

**DOI:** 10.1016/j.dib.2015.12.007

**Published:** 2015-12-17

**Authors:** Christian W. Johnson, Greg Buhrman, Pamela Y. Ting, John Colicelli, Carla Mattos

**Affiliations:** a Department of Chemistry and Chemical Biology, Northeastern University, Boston, MA 02115, USA; b Department of Molecular and Structural Biochemistry, North Carolina State University, Raleigh, NC 27695, USA; c Molecular Biology Institute, Jonsson Comprehensive Cancer Center, Department of Biological Chemistry, David Geffen School of Medicine, University of California, Los Angeles, CA, USA

**Keywords:** RAS GTPase, Protein purification, Nucleotide exchange, X-ray crystal structures

## Abstract

This article expands on crystal structure data for human H-RAS with mutations at position Y137, briefly described in a paper on the effects of phosphorylation of Y137 by ABL kinases (Tyrosine phosphorylation of RAS by ABL allosterically enhances effector binding, published in the FASEB Journal [1]). The crystal structures of the Y137E mutant (phosphorylation mimic) and of the Y137F mutant (without the hydroxyl group where phosphorylation occurs) were deposited in the Protein Data Bank with PDB codes 4XVQ (H-RAS^Y137E^) and 4XVR (H-RAS^Y137F^). This article includes details for expression and purification of RAS and its mutants with no affinity tags*,* in vitro exchange of guanine nucleotides, protein crystallization, X-ray data collection and structure refinement.

**Specifications Table**

**Table:** 

Subject area	Biology
More specific subject area	Structural biology
Type of data	Molecular models obtained by protein X-ray crystallography
How data was acquired	X-day diffraction data on the protein crystals were obtained using a HighFlux HomeLab consisting of a Rigaku MicroMax-007HF, a second generation microfocus rotating anode generator with a 70 μm diameter focal spot. The X-ray generator is mounted on a R-Axis IV++ image plate detector with VariMaxHR optics.
Data format	X-ray data processed with HKL3000
Experimental factors	X-ray data were collected at a temperature of 100 K and with a crystal to detector distance of 120 mm.
Experimental features	Protein purification did not involve affinity tags. The reservoir solution in the crystallization plates contained a small amount of the buffer solution present in the crystallization drop. Crystals were cryo-protected with crystallization solution containing 30% glycerol prior to freezing in liquid nitrogen.
Data source location	Boston, MA United States
Data accessibility	Data were deposited in the Protein Data Bank (PDB). The accession codes are PDB: 4XVQ and PDB: 4XVR.

**Value of the data**

• Structural data on RAS are essential for understanding its function.
• The methods we used to study RAS can in general elucidate the links between mutations at an allosteric site and catalysis at the active site of proteins.
• The protocols presented here can be used in general to obtain the structure of any RAS mutant.

## 1 Data, experimental design, materials and methods

The experimental protocols presented here focus on data collection for the structural analysis of RAS proteins and their mutants. RAS exists in three major isoforms, H-, K- and N-RAS, which together are found mutated in about 20% of all human cancers. While the most common mutation sites are at residues G12, G13 and Q61, mutants at other sites are also found in human tumors [2]. The type of data presented here is important for studying oncogenic mutants, but they are also essential for making mutants that test the relevance of particular structural features to the function of RAS, as was the case for the allosteric switch mutants that we recently published [1]. The present article provides details on how to express and purify any of the RAS isoforms and their mutants with a truncated C-terminal hypervariable region.

Mutagenesis, protein purification, and crystallization were done using the G-domain (residues 1–166) of wild type H-RAS (EC 3.6.5.2) inserted into the pET21 vector system. Primer design for mutagenesis was done according to the QuikChange® site-directed mutagenesis protocol. The Y137E mutant was made using parameters recommended by the QuikChange® manual. The Y137F mutant was made using a previously published two-step mutagenesis procedure [3]. Targeted mutagenesis was confirmed by third-party sequencing (Eurofins MWG-Operon). Plasmids containing the cDNA for H-RAS^Y137E^ and H-RAS^Y137F^ were used to transform *Escherichia coli*, strain BL21, in order to express and purify the mutant proteins.

For protein expression, 200 mL of Luria Broth (LB) containing ampicillin (50 mg/mL) was inoculated with transformed BL21 cells and grown overnight at 37 °C. On the following day, six Fernbach flasks, each with 1 L of LB containing ampicillin, were inoculated with 25 mL of the overnight culture. Cell growth was monitored until the optical density (O.D.) reached 0.6 (80–120 min), as measured by a SmartSpec™ Plus spectrophotmeter. Protein expression was then induced by adding dry IPTG to a final concentration of 0.5 mM in each liter of growing *E. coli*. The temperature was reduced to 32 °C, and protein expression was allowed to continue for six hours. Cultures were shaken at 225 rpm during both the cell growth and protein expression periods. After six hours, the cells were pelleted by centrifugation at 7000 rpm for 20 min at 4 °C and the cell pellet stored at −80 °C.

For protein purification, cells were solubilized (200–300 mg of cells/mL) in buffer A (20 mM Tris, pH 8.0, 5 mM MgCl_2_, 50 mM NaCl, 5% glycerol, 20 µM GDP, 1 mM DTT) containing benzamidine (40.0 µM), leupeptin (0.17 µM), and antipain (0.12 µM). H-RAS^Y137F^ suspension also contained pefabloc (8.0 µM). Cells were lysed by sonication (60 Sonic Dismembrator from Fisher Scientific) for 30 s at 18 W, followed by 30 s of rest, on ice in a metal cup. Five rounds of sonication were performed. The sonicated lysate was clarified by centrifugation at 14,000 rpm for 20 min at 4 °C. Polyethyleneimine (PEI, 0.02% w/v) was then used to precipitate contaminating nucleic acids and proteins [4] in the lysate supernatant. Precipitation of unwanted macromolecules with PEI was done on ice, while the lysate solution was stirred gently for 30 min. After precipitation, clarification of lysate was again performed by centrifugation at 14,000 rpm for 20 min at 4 °C. Just prior to chromatography, the protein solution was passed through a 0.45 µm pore membrane using syringe filtration to remove remaining cell debris and large protein aggregates.

**Fig. 1:**
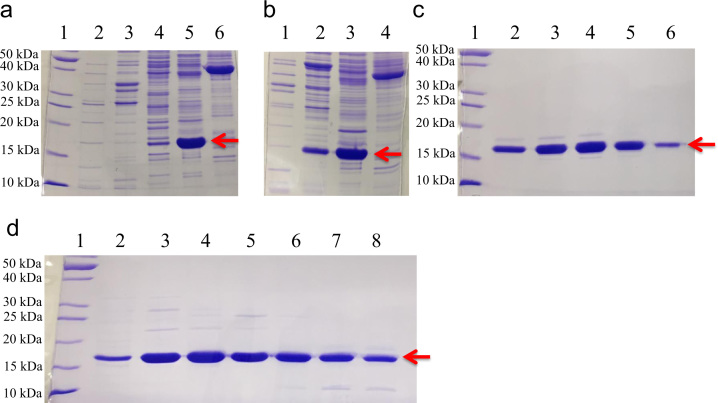
15% SDS-PAGE gels of generalized purification of H-Ras Y137F and E. Purifications of Y137F and Y137E are very similar. Lanes represent fractions collected from anion exchange chromatography and gel filtration. Fractions from anion exchange are described in terms of percentage of buffer B and column volume (cv); fractions for gel filtration are in terms of cv only. Panel (a) shows results from QFF of H-Ras Y137E. Lanes for panel (a) are: (1) MW ladder, (2) 7% B and 7.7 cv, (3) 11% B and 9.1 cv, (4) 16% B and 10 cv, (5) 18% B and 11 cv, and (6) 23% B and 13 cv. Panel (b) shows results from QFF of H-Ras Y137F. Lanes for panel (b) are: (1) 8.5% B and 8.5 cv, (2) 13% B and 9.9 cv, (3) 17% B and 11 cv, and (4) 23% B and 13 cv. Panel (c) shows results of gel filtration of H-Ras Y137F. Lanes for panel (c) are: (1) MW ladder, (2) 0.51 cv, (3) 0.53 cv, (4) 0.54 cv, (5) 0.55 cv, and (6) 0.57 cv. Panel (d) shows results for QHP of H-Ras Y137E. Lanes for panel (d) are: (1) MW ladder, (2) 14.5% B and 20.3 cv, (3) 14.8% B and 29.8 cv, (4) 15.3% B and 30.6 cv, (5) 15.8% B and 31.4 cv, (6) 16.2% B and 32.2 cv, (7) 16.7% B and 33.0 cv, and (8) 16.8% B and 33.8 cv. The red arrow depicts the H-Ras mutant (~18.8 kDa), and the markers for the ladder are shown on the left of gels (a), (c), and (d).

Chromatography for purification of H-RAS^Y137E^ and H-RAS^Y137F^ was done using a ÄKTA FPLC system (GE Healthcare) at 4 °C. The first step was anion exchange (HiPrep™ 16/10 QFF column, 20 mL column volume (cv), GE Healthcare). Binding of mutant H-RAS to a QFF column was done in buffer A without protease inhibitors and at a flow rate of 4 mL/min. After protein binding, the column was washed with two column volumes of buffer A. Protein elution was performed using a 200 mL gradient of 0–40% buffer B (20 mM Tris, pH 8.0, 5 mM MgCl_2_, 1 M NaCl, 5% glycerol, 20 µM GDP, 1 mM DTT) at 4 mL/min. Fig. 1a and b shows SDS-PAGE of sample fractions coming off the QFF column as the gradient increases. The two mutants are very similar in terms of their migration in this column. H-Ras^Y137E^ and H-Ras^Y137F^ eluted off the QFF column at around 15–25% buffer B, which is common for RAS and its mutants. Only the fractions containing a substantial amount of RAS protein were collected for further purification. The second chromatography step was size exclusion (HiPrep™ 26/60 Sephacryl S-100 or S-200 column, 320 mL cv, GE Healthcare) using Buffer A at a rate of 1 mL/min. Fig. 1c shows an example of the gel filtration fractions containing H-RAS^Y137F^. This mutant was pure enough at this stage to be used for crystallization. Gel filtration was also performed for H-RAS^Y137E^ mutant (results are not shown). Some mutants, such as H-RAS^Y137E^, require an additional purification step. For this we use a higher resolution, anion exchange chromatography column (HiTrap™ QHP column, 5 mL cv, GE Healthcare). The Y137E protein was bound to the QHP column using buffer A at 4 mL/min. After protein binding, the column was washed with 4 column volumes of buffer A. Protein was eluted from the QHP column using 0–25% buffer B over 200 mL, at a rate of 4 mL/min. Fig. 1d shows the Y137E mutant RAS protein eluted from the QHP column, pure enough to be used for crystallization.

**Fig. 2:**
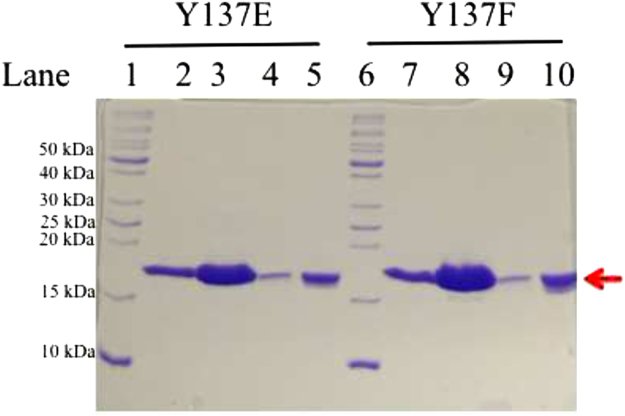
SDS-PAGE showing purity of H-RAS^Y137F^ and H-RAS^Y137E^. Left side of 15% SDS-PAGE shows H-RAS^Y137E^ and right side shows H-RAS^Y137F^. The 10 lanes on the gel represent the following: MW ladder (lanes 1 and 6), H-RAS^Y137E^ bound to GDP (lane 2), H-RAS^Y137E^ bound to GppNHp (lane 3), 5-fold dilution of H-RAS^Y137E^ bound to GDP (lane 4), 5-fold dilution of H-RAS^Y137E^ bound to GppNHp (lane 5), H-RAS^Y137E^ bound to GDP (ladder 7), H-RAS^Y137E^ bound to GppNHp (lane 8), 5-fold dilution of H-RAS^Y137E^ bound to GDP (lane 9), and 5-fold dilution of H-RAS^Y137E^ bound to GppNHp (lane 10). The red arrow depicts the H-Ras mutant (~18.8 kDa), and the markers for the MW ladder are shown on the left.

In general, RAS proteins are purified bound to GDP. This was the case for both H-RAS^Y137E^ and H-RAS^Y137F^. However, our interest is in the GTP-bound active state. Since GTP is hydrolyzed to GDP in the experimental time frame for structure determination we loaded RAS with the GTP analog guanylyl-imidodiphosphate (GppNHp). Nucleotide exchange of GDP for GppNHp was performed by first transferring the protein into Nucleotide Exchange (NE) buffer (32 mM Tris pH 8.0, 200 mM ammonium sulfate, 10 mM DTT, and 0.15% N-octylglucopyranoside) using Illustra™ Sephadex™ DNA grade gravity columns (GE Healthcare), following the manufacture׳s instructions. Once exchanged into NE buffer, 1 mg of GppNHp, and 50 U of alkaline phosphatase linked to agarose beads (Sigma-Aldrich®) were added per 10 mg of protein. Protein was then gently rotated for 30–60 min at 37 °C. After nucleotide exchange, a concentrated stock solution of MgCl_2_ was used to bring the protein solution to a final concentration of 20 mM MgCl_2_. After 5 min at room temperature, the protein solution was exchanged into a stabilization buffer (20 mM HEPES, pH 7.5, 50 mM NaCl, 20 mM MgCl_2_, 1 or 10 mM DTT). The protein was then concentrated, flash frozen, and stored at −80 °C until it was used for crystallization. Fig. 2 shows SDS-PAGE for the purified H-RAS^Y137E^ and RAS^Y137F^ proteins prior to crystallization.

**Fig. 3:**
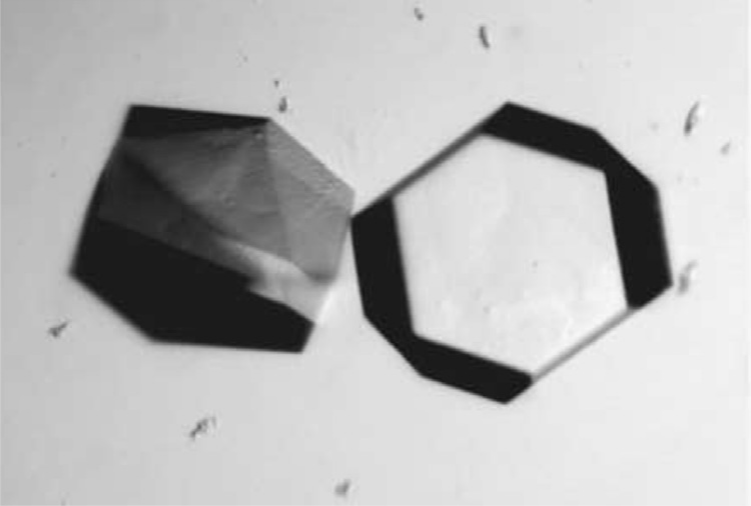
Crystals of wild type H-RAS with symmetry of space group P3_2_21.

All reagents and materials for crystallization were purchased from Hampton Research, Inc. Crystals were grown in 24-well plates with reservoir volumes ranging from 425 µL to 625 µL. H-RAS^Y137E^ was crystallized in hanging drops containing 2 µL protein at a concentration of 12.4 mg/mL in stabilization buffer and 2 µL of reservoir solution consisting of 152 mM Ca(OAc)_2_, 24.8% PEG 3350, and 4.8% stabilization buffer at pH 7.5. For H-RAS^Y137F^ crystallization, the protein was at a concentration of 20.7 mg/mL and the reservoir contained 139 mM Ca(OAc)_2_, 22.6% PEG 3350, and 13% stabilization buffer at pH 7.5. Both proteins crystallized with symmetry of space group P3_2_21. Fig. 3 shows crystals of wild type H-RAS in this crystal form. The mutant crystals look similar. Data for H-RAS^Y137E^ and H-RAS^Y137F^ were collected using a Rigaku X-ray generator at a temperature of 100 K with X-ray wavelength of 1.54 Å. Data were processed with HKL2000 [5]. The coordinates with PDB code 1CTQ were used as a phasing model for molecular replacement, followed by refinement using the PHENIX suite of programs [6] and model building using COOT [7]. Several rounds of refinement were done, starting with the protein model, followed by subsequent addition of the GppNHp molecule and crystallographic water molecules. The table of data collection and structure refinement statistics is found in the original publication, as are figures showing the structures of the mutant Ras proteins [1].

## Conflicts of interest

None
